# 1741. A Phase 4, Open-label, Multicenter Study to Evaluate the Safety, Tolerability, and Immunogenicity of Vaxelis™ in Healthy Children Previously Vaccinated With a 2-Dose Primary Infant Series of Either Vaxelis™ or Hexyon™ (V419-016)

**DOI:** 10.1093/ofid/ofad500.1572

**Published:** 2023-11-27

**Authors:** Andrea Guerra, Claudio Costantino, Federico Martinon-Torres, Soeren Westerholt, Courtney Lambeth, Ziqiang Chen, Jessie Lumley, David Johnson, Marissa B Wilck

**Affiliations:** Merck Sharp & Dohme LLC - United Kingdom, London, England, United Kingdom; University of Palermo, Palermo, Sicilia, Italy; Hospital Clínico Universitario de Santiago, Santiago de Compostela, Spain, Santiago de Compostela, Galicia, Spain; Praxis fur Kinder-und Jugendmedizin Standort Vorsfelde, Wolfsburg, Sachsen, Germany; Merck & Co., Inc., Rahway, New Jersey; Merck & Co., Inc., Rahway, New Jersey; Merck & Co., Inc., Rahway, New Jersey; Sanofi Pasteur, Swiftwater, PA; Merck & Co., Inc., Rahway, New Jersey

## Abstract

**Background:**

Vaxelis™ is a hexavalent combination vaccine (HCV) indicated in infants and toddlers for the prevention of diphtheria, tetanus, pertussis, hepatitis B, poliomyelitis, and invasive disease due to *Haemophilus influenzae* type b. In practice, switching between HCVs during the childhood vaccination series is sometimes necessary due to vaccine availability, health care provider preference, tender awards, or other reasons. The various HCVs differ in the types of antigens used, which may affect immunogenicity and reactogenicity. The use of Vaxelis™ as a booster dose after another HCV has not previously been studied in a clinical trial. The purpose of this study was to describe the safety, tolerability, and immunogenicity of a booster dose of Vaxelis™ in toddlers who previously received a primary infant series of either Vaxelis™ or Hexyon™.

**Methods:**

Healthy participants approximately 11 to 13 months of age who previously received a two-dose primary series of Vaxelis™ (VVV group) or Hexyon™ (HHV group) at approximately 2 and 4 months of age all received a Vaxelis™ booster dose. Safety was evaluated as the proportion of participants with adverse events (AEs). Immunogenicity was evaluated by measuring antibody levels to individual vaccine antigens approximately 30 days following booster vaccination.

**Results:**

Participant characteristics were comparable between groups (VVV group n=85; HHV group n=82). The overall proportions of participants with AEs­─including injection-site, systemic, vaccine-related, and serious AEs─were generally comparable between groups. The proportions of participants with antibody-specific responses for antigens contained in both Vaxelis™ and Hexyon™ at 30 days postvaccination with Vaxelis™ were comparable between groups and higher in the VVV group for antigens FIM2/3 and PRN found only in Vaxelis™ (Table 1).

**Table 1.**
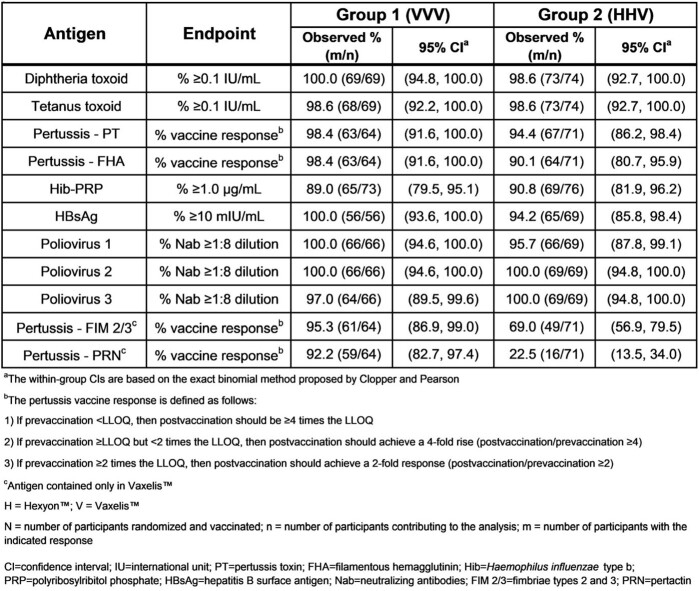
Proportions of participants meeting specified Vaxelis™ antigen responses at 30 days postvaccination

**Conclusion:**

A booster dose of Vaxelis™ was well tolerated following a primary series of Hexyon™ with a safety profile similar to a 3-dose series of Vaxelis™. Immune responses were comparable for all shared antigens and higher for those found only in Vaxelis™. These data support the use of Vaxelis™ as a booster in mixed HCV regimens.

**Disclosures:**

**Andrea Guerra, MD**, Merck Sharp & Dohme LLC - United Kingdom: employee|Merck Sharp & Dohme LLC - United Kingdom: Stocks/Bonds **Federico Martinon-Torres, MD, PhD, Assoc. Prof**, Ablynx, Gilead, Regeneron, Roche, Abbott and MedImmune: Honoraria|Ablynx, Gilead, Regeneron, Roche, Abbott and MedImmune: principal investigator|Astra Zeneca, GSK, Pfizer Inc, Sanofi, MSD, Seqirus, Moderna, Novavax, Biofabri, and Janssen: Advisor/Consultant|Astra Zeneca, GSK, Pfizer Inc, Sanofi, MSD, Seqirus, Moderna, Novavax, Biofabri, and Janssen: Board Member|Astra Zeneca, GSK, Pfizer Inc, Sanofi, MSD, Seqirus, Moderna, Novavax, Biofabri, and Janssen: Honoraria|Astra Zeneca, GSK, Pfizer Inc, Sanofi, MSD, Seqirus, Moderna, Novavax, Biofabri, and Janssen: speaker and investigator **Courtney Lambeth, BS**, Merck Sharp & Dohme LLC, a subsidiary of Merck & Co., Inc., Rahway, NJ, USA: employee|Merck Sharp & Dohme LLC, a subsidiary of Merck & Co., Inc., Rahway, NJ, USA: Stocks/Bonds **Ziqiang Chen, PhD**, Merck Sharp & Dohme LLC, a subsidiary of Merck & Co., Inc., Rahway, NJ, USA: employee|Merck Sharp & Dohme LLC, a subsidiary of Merck & Co., Inc., Rahway, NJ, USA: Stocks/Bonds **Jessie Lumley, MA**, Merck Sharp & Dohme LLC, a subsidiary of Merck & Co., Inc., Rahway, NJ, USA: employee|Merck Sharp & Dohme LLC, a subsidiary of Merck & Co., Inc., Rahway, NJ, USA: Stocks/Bonds **David Johnson, MD, MPH**, Sanofi: employee **Marissa B. Wilck, MD**, Merck Sharp & Dohme LLC, a subsidiary of Merck & Co., Inc., Rahway, NJ, USA: employee|Merck Sharp & Dohme LLC, a subsidiary of Merck & Co., Inc., Rahway, NJ, USA: Stocks/Bonds

